# Fabrication of Multifunctional Silylated GO/FeSiAl Epoxy Composites: A Heat Conducting Microwave Absorber for 5G Base Station Packaging

**DOI:** 10.3390/ma16247511

**Published:** 2023-12-05

**Authors:** Zhuyun Xie, Dehai Xiao, Qin Yu, Yuefeng Wang, Hanyi Liao, Tianzhan Zhang, Peijiang Liu, Liguo Xu

**Affiliations:** 1 Centre of Chip Chemistry, Huangpu Institution of Materials, Changchun Institute of Applied Chemistry, Chinese Academy of Sciences, Guangzhou 510663, China; zx263@cornell.edu (Z.X.); dhxiao@ciac.ac.cn (D.X.); y187459762379@163.com (Q.Y.); wyf@mail.ipc.ac.cn (Y.W.); liaohanyi@ciac.ac.cn (H.L.); 2 College of Material Science and Engineering, Jilin Jianzhu University, Changchun 130119, China; zhangtz048@mail.ipc.ac.cn; 3 Reliability Physics and Application Technology of Electronic Component Key Laboratory, The Fifth Electronics Research Institute of the Ministry of Information Industry, Guangzhou 510610, China; 4 College of Light Chemical Industry and Materials Engineering, Shunde Polytechnic, Foshan 528333, China

**Keywords:** wave absorption, thermal conduction, 5G base station, packaging materials, epoxy resin

## Abstract

A multifunctional microwave absorber with high thermal conductivity for 5G base station packaging comprising silylated GO/FeSiAl epoxy composites were fabricated by a simple solvent-handling method, and its microwave absorption properties and thermal conductivity were presented. It could act as an applicable microwave absorber for highly integrated 5G base station packaging with 5G antennas within a range of operating frequency of 2.575–2.645 GHz at a small thickness (2 mm), as evident from reflection loss with a maximum of −48.28 dB and an effective range of 3.6 GHz. Such a prominent microwave absorbing performance results from interfacial polarization resonance attributed to a nicely formed GO/FeSiAl interface through silylation. It also exhibits a significant enhanced thermal conductivity of 1.6 W/(mK) by constructing successive thermal channels.

## 1. Introduction

With the popularization of 5G communication, 5G base stations are increasingly distributed. The 5G base station has a higher frequency band, an ultra large bandwidth, more transmitting and receiving antennas, and more complex beamforming working modes compared to 4G applications, which requires increasing power density and integration [1]. This may cause a series of problems. On one hand, a number of digital parts inside the 5G base station, such as high-frequency signal lines, pins of integrated circuits, and various types of connectors, may emit mass microwaves, affecting the normal operation of microwave-sensitive elements inside and outside the 5G station [2,3]. It may also have an interaction with living species [4]. On the other hand, higher power density generates more heat. This excess heat has difficulty exporting through thermal conduction between electronic components, causing the temperature inside the base station to rise, significantly reducing its operating life [5]. Therefore, the demand for heat-conducting and wave-absorbing materials is growing rapidly. To solve this problem, thermally conductive materials are applied on the surface of electronic components. However, since thermal conductive material already occupies limited space inside of the device gap, there is no space for additional wave-absorbing material [6]. Also, the continuous thermal path required for heat conduction will be drastically reduced once it is blocked by wave-absorbing materials with low thermal conductivity [7]. Therefore, the demand for a material with both heat-conducting and wave-absorbing properties is growing rapidly.

**Figure 1:**
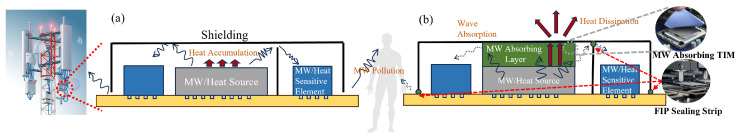
5G base station (**a**) without wave-absorbing and heat conducting measure, (**b**) with wave absorbing layers and FIP sealing strips.

Figure 1 shows the typical application and working mechanism of existing wave-absorbing materials in 5G base stations. There are two widely used solutions—one is microwave-absorbing thermal interface material (TIM) [8] by attaching a functional polymer pad directly to the surface of the microwave or heat source so that the heat can be directly transmitted to the shielding and dissipated. The second method is the form-in-place (FIP) sealing gasket [9], which acts in the gaps at bulkhead joints and the gaps at shielding joints to avoid the leakage of electromagnetic waves and the blocking of heat conduction. Leading product providers include Laird Co., Nolato Co., FRD Co., etc. However, existing products either have a relatively low reflection loss of −10 dB with a low thermal conductivity of 1 W/(mK) or have a high thermal conductivity but with no wave-absorbing ability [10]. A material that combines both properties has yet to be developed. The most widely used packaging materials for the 5G base station are polymer materials, among which epoxy resin attracts attention for its excellent adhesive and mechanical properties. However, epoxy resins have poor thermal conductivity and microwave-absorbing properties [11,12]. Normally, functional fillers are used in polymer materials to achieve better thermal and magnetic properties. Recently, carbon materials have received extensive attention thanks to their excellent dielectric properties as well as high electrical conductivity, such as carbon nanotubes, graphene, etc. [13,14,15,16,17,18]. Among them, graphene oxide has residual defects and an amount of epoxy, hydroxyl, and carboxyl groups on its surface. These factors may result in the transition from contiguous states to the Fermi level, thus proposing an impedance match performance. Moreover, defect polarization relaxation and groups’ electronic dipole polarization relaxation are also beneficial for enhancing their wave-absorbing performance [19]. Last but not least, materials with 2D structures, such as graphene and MXene, exhibit excellent thermal conducting and wave-absorbing properties. Sun et al. [20] reported a self-assembly anchored MXene nanosheet loaded with CMWCNTs with a maximum reflection loss of −46 dB at a thickness of 1.5 mm. Graphene has a thermal conductivity of 5000 W/(mK) in the plane direction and 30 W/(mK) in the longitudinal direction [21].

Yet, simply using graphene oxide as a thermally conductive and wave-absorbing filler for packaging material is impractical for several reasons. Firstly, due to its large specific surface area, a very small amount of graphene oxide will have a huge impact on the fluidity of the compounds [22]. It has been reported that pure graphene filling can only achieve a maximum of −7 dB of reflection loss [19]. Secondly, graphene oxide, working as a dielectric loss absorber and exhibiting a high reflection of microwaves, may emit microwave pollution to the surroundings [22]. To improve this, many attempts have been made to combine GO with magnetic particles. Zou et al. [23] developed Fe/GO nanocomposites by inserting Fe${}^{3+}$ into GO followed by a reduced reaction in $H_{2}$. The maximum reflection loss of 9 dB was at 11–18 GHz. Li et al. [24] synthesized GO/Fe${}_{3}$O${}_{4}$/ iron phthalocyanine composites using a facile one-step solvothermal method. They observed the maximum microwave absorption of −27.92 dB at 10.8 GHz. Ghosh et al. [25] fabricated n-doped GO/MnCo${}_{2}$O${}_{4}$ nanocomposites using a facile hydrothermal method followed by an annealing process, and the reflection loss was observed to fall in the range of −90 to −77 dB.

Nevertheless, the synthesis processes of the above studies are not practical for scaling up production. Selecting a suitable magnetic particle and finding a feasible way to produce the compound is important for rapid application. FeSiAl, as a type of soft magnetic alloy, has become widely used in the microwave absorption field for its excellent magnetic properties such as high saturation magnetization and high eddy current loss causing by its high permeability in the low-frequency range [26,27,28]. However, its high conductivity, along with a low Snoek limit at a high frequency, limit its application [29,30,31].

In this study, a novel epoxy-based 5G base station packaging material with comprehensive properties of high wave-absorbing ability and high thermal conductivity ability was introduced, in which GO/FeSiAl particles were made and modified by γ-aminopropyl triethoxysilane in gentle solvent conditions. By combining these two particles, an impedance match can be formed, improving its high-frequency range wave-absorbing ability [30]. Meanwhile, graphene has a large specific surface area and can act as an anti-settling agent [32], providing another application advantage. Our work provides a simple and practical way to produce multifunctional 5G base station packaging materials for application at a large scale, which has an advanced reflection loss (RL) of −48.28 dB and a wide effective range of 3.6 GHz within the range of operating frequency of 5G antennas of 2.575–2.645 GHz at a small thickness (2 mm).

## 2. Materials and Methods

### 2.1. Materials

Graphene powder was purchased from XFnano Inc. (Nanjing, China) and N-butylamine (99.7%) was purchased from Macklin (Shanghai, China). γ-aminopropyl triethoxysilane was purchased from Changhe Chemical Co., (Hangzhou, China), FeSiAl was purchased from Mana New Material Co., (Changsha, China), Epoxy resin (BE 188EL) was purchased from Changchun Chemical Co., (Taipei, Taiwan), and Polyetheramine (Jeffamine D-2000) was purchased from Huntsman Corp., (The Woodlands, TX, USA).

### 2.2. Preparation of GO/Ethanol Suspension

The GO was prepared by oxidizing graphene powder using the Hummers’ method [33]. After oxidation, the obtained graphene oxide powder was washed several times using deionized water through a centrifuge to remove the residual salts and acids. The GO was then silylated according to Mastsuo’s method [34]. The washed GO was mixed with a certain amount of butylamine for exfoliation and then subjected to ultrasonication for 30 min. The dispersion was refluxed at 60 °C for 60 min. Then, the exfoliated GO was centrifuged with ethanol several times to remove the residual butylamine and was dispersed into ethanol via ultrasonication for 30 min.

### 2.3. Preparation of Silylated GO@FeSiAl Nanoparticles

A certain amount of FeSiAl powder was added into GO/ethanol suspension at different ratios. The weight ratio of FeSiAl and GO is listed in Table 1, and varied from 1000:1 to 10:1. Then, the GO/FeSiAl was dispersed into an ethanol/deionized water solution (0.1 g/mL), in which the weight ratio of ethanol and deionized water was 9:1. The mixture was stirred and refluxed for 3 h at 80 °C, while slowly dripping γ-aminopropyl triethoxysilane (0.2 mg/mL). The silylated GO/FeSiAl was suction-filtrated and vacuum dried at 80 °C for 24 h. A group of pure GO without FeSiAl was created as the control group compared to the GF groups. GO-1 was the GO having gone through all the processes but without FeSiAl.

### 2.4. Preparation of GO/FeSiAl Epoxy Compounds

The dried GO/FeSiAl powder was then mixed with epoxy resin and polyetheramine using a planetary stir at a speed of 2000 rpm for 5 min, in which the active hydrogen equivalent of epoxy resin and polyetheramine was 1:1. Then, the epoxy resin compound was cured at 80 °C for 24 h. The resulting sample was called a GO/FeSiAl epoxy compound.

### 2.5. Characterization Techniques

The scanning electron microscope (SEM) images of particles were taken on a JSM-IT800 instrument, JEOL, Akishima-shi, Tokyo, Japan operating with 5 kV. The X-ray diffraction (XRD) patterns of the compounds were acquired in the range of 10–90° on a D8 ADVANCE instrument, Bruker, Billerica, MA, USA with CuKα radiation and a scanning rate of 3°/min. The Fourier transform infrared spectroscopy (FTIR) analysis of particles was recorded in KBr in the form of a compressed pellet in the range of 400–4000 cm${}^{-1}$ on Vertex 70 V. Bruker, Billerica, MA, USA.

**Figure 2:**
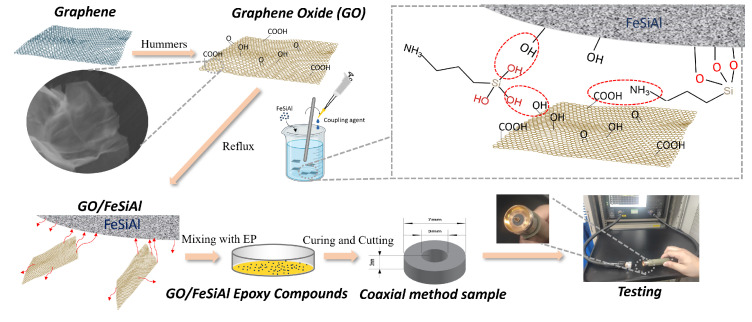
Schematic illustration for the fabrication of GO/FeSiAl epoxy compounds and testing methods.

Microwave absorption properties were characterized by measuring the magnetic and dielectric properties of the compounds in the frequency range from 0.1 GHz to 18 GHz, covering most of the microwave pollution frequency in our daily life, such as communication devices, satellite communications, radar, etc. [35]. The sample was prepared in the shape of concentric circles with a thickness of 3 mm, an inner diameter of 3 mm, and an outer diameter of 7 mm, and was tested using the coaxial method based on the ASTM D5568-2a standard [36], on an ENA Series Network Analyzer, N5080a Agilent, Santa Clara, CA, USA. As shown in Figure 2, the concentric circle sample was put on the coaxial airline (sample holder) and then accessed the coaxial transmission line through a connector to the Network Analyzer. Then, the reflection loss was calculated using transmission line theory. A detailed illustration of the fabrication and the characterization method is presented in Figure 2.

## 3. Results and Discussions

### 3.1. Morphology and Structural Analysis

**Figure 3:**
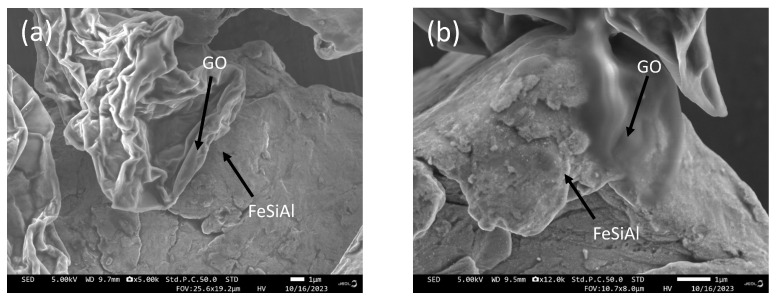
SEM images of (**a**) GF-0 and (**b**) GF-3.

SEM images of GO/FeSiAl composites with or without a coupling agent are displayed in Figure 3. GF-3 presents a blurred interface of GO and FeSiAl, suggesting a good compatibility, whereas GF-0 shows a clear interface, indicating poor compatibility. It can be concluded that the use of coupling agents enhances the compatibility of GO and FeSiAl particles, which is important for the generation of heterogeneous interfaces and a stronger interfacial polarization loss.

**Figure 4:**
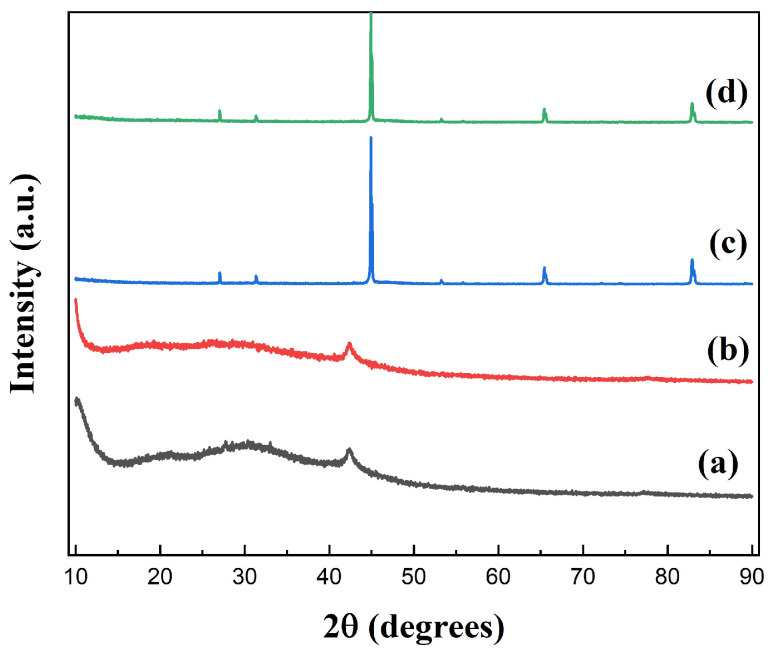
XRD spectra of (a) GO, (b) silylated GO, (c) FeSiAl, and (d) GF-3.

Figure 4 shows the XRD spectra of GO, GO-1, FeSiAl, and GF-3. The diffraction peak at 2θ = 11° derived from GO shifted to the lower region in GO-1, indicating that the interlayer space increased during the silylating process. It might be caused by long coupling agent molecules attaching to the GO sheet surface and exfoliating the GO sheets, causing the interlayer spacing to increase. In Figure 4 (c), there are three peaks associated with (220), (400), and (211) planes, consisting of the standard diffraction spectra of the body-centered cubic structure, FeSiAl. There is a fairly gentle peak within the range of 2θ = 20° to 2θ = 35° of GF-3 (Figure 4 (d)) compared to FeSiAl, which is the characteristic peak of GO, indicating that a small amount of GO was grafted onto FeSiAl.

**Figure 5:**
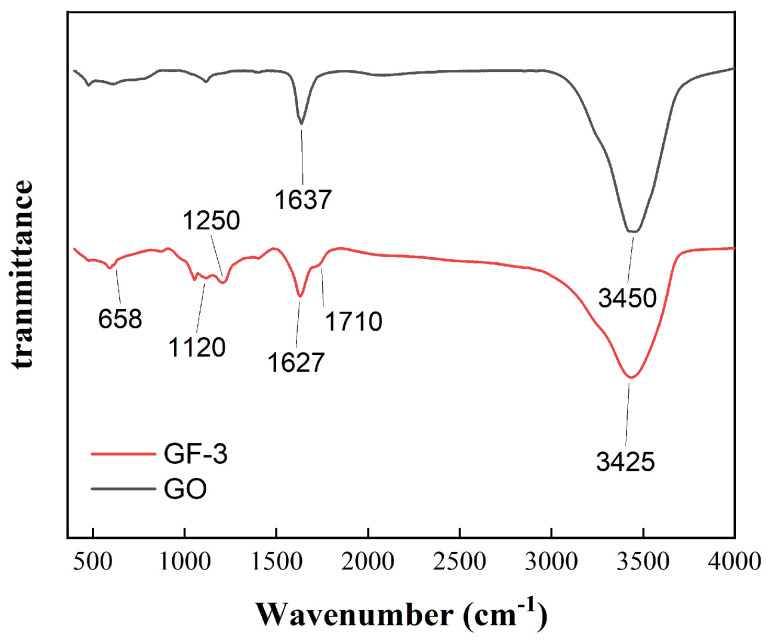
FTIR spectra of GO and GF-3.

Figure 5 shows the FTIR spectra of the GF-0 and GF-3 samples. The absorption peak of GF-0 at 3450 cm${}^{-1}$ is attributed to -OH from absorbing water and hydrogen bonds between the layered structure of GO. In addition, the absorption peak of GF-3 at around 3450 cm${}^{-1}$ shifts to 3425 cm${}^{-1}$ and is lower than that of GO, indicating that the force between GO layers is weakened and chemical bonding between GO and FeSiAl is generated instead. The relative intensity of the peaks at 1637 cm${}^{-1}$ is derived from the C=O group, which represents functional groups on the GO surface and becomes wider in GF-3, resulting from several peaks overlapping each other. Those peaks are generated from the amide groups by the carboxyl group on the GO surface reacting with the amino group on the coupling agent. The peak at 1250 cm${}^{-1}$ represents the stretching and bending vibration of C-N groups in the amide group or coupling agent. Moreover, the peaks around 1120 cm${}^{-1}$ and 1250 cm${}^{-1}$ are owing to the Si-O bond and the stretching and bending vibration of the C-N groups overlapping each other, which may be from the amide group, products of the coupling agent and epoxy groups, or the coupling agent itself. The peak at 658 cm${}^{-1}$ is owing to the stretching vibration of N-H groups from amide or amino groups. Since FeSiAl is a metallic compound and shows little or no transmittance, these results suggest that the coupling agent was successfully grafted on GO.

### 3.2. Microwave Absorption Properties

To relieve the microwave absorption mechanism of the GO/FeSiAl/epoxy compound, the influence of GO amounts on reflection loss, impedance matching, and the attenuation constant was studied.

**Figure 6:**
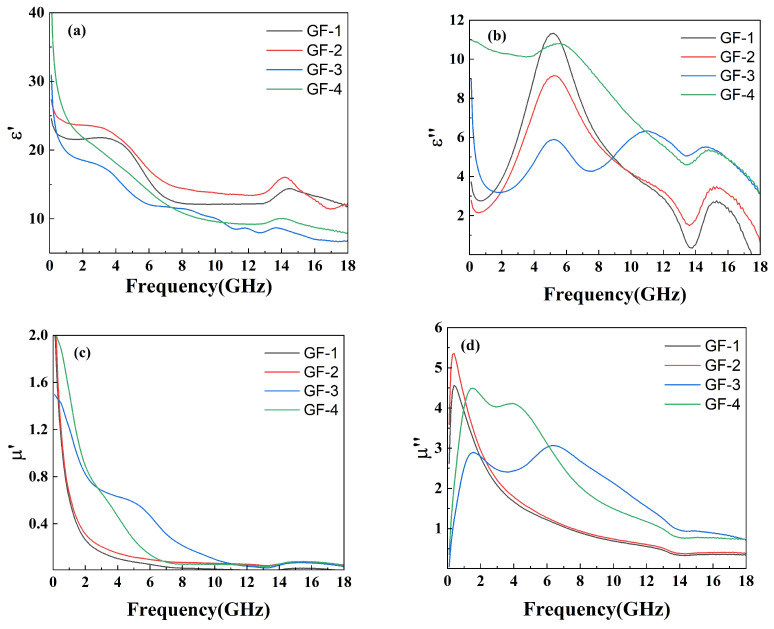
Plots of (**a**) ε${}^{\prime }$, (**b**) ε${}^{\prime \prime }$, (**c**) μ${}^{\prime }$, and (**d**) μ${}^{\prime \prime }$ versus frequency for GO/FeSiAl epoxy compounds with different GO amounts.

The electromagnetic properties were tested through the coaxial method within the frequency range of 0 GHz to 18 GHz to reveal the relationship between the complex permeability real part (μ${}^{\prime }$), the complex permeability imaginary part (μ${}^{\prime \prime }$), the relative complex permittivity real part (ε${}^{\prime }$), the relative complex permittivity imaginary part (ε${}^{\prime \prime }$), and the amount of GO in the compounds, as shown in Figure 6.

Figure 6a shows a decreasing trend of ε${}^{\prime }$ with increasing GO amounts, responding to the ability of the material to store charge decreases. This can be explained through the micro-capacitance model [37]. Neighboring FeSiAl presented in the matrix can be seen as two flat plates of micro-capacitor. While GO conducts electricity very well and has poor ability to stores electrons, therefor cannot form a micro-capacitor structure within itself or with FeSiAl [38]. Thus, the addition of GO will destroy the micro-capacitor between the FeSiAl and leads to a decrease in charge storing ability. In Figure 6b, the ε${}^{\prime \prime }$ values exhibit an obvious peak in sample GF-1 and sample GF-2 with fewer or no GO. As the amount of GO increases, more peaks appear at higher frequencies. These peaks can be ascribed to the interfacial polarization resonance, indicating more GO-FeSiAl interface in the system as well as defects and functional groups on the surface of GO resulting in higher interfacial polarization. This contributes to higher microwave absorption at higher frequencies [39,40]. As the frequency rises, μ${}^{\prime }$ shows a collectively decreasing trend (Figure 6c). This may be explained by high-frequency fields resulting in polarization hysteresis and dielectric relaxation. This trend slows down as more GO is added. The complex permeability imaginary part (ε${}^{\prime \prime }$) of GF-1 and GF-2 with a lower amount of GO presents a pronounced and sharp peak at 0.3 GHz, which is attributed to the magnetic capacity of the FeSiAl consuming electromagnetic energy, as shown in Figure 6d. The high-frequency shift to 2 GHz and widening of peaks for samples with a higher GO amount may be ascribed to the suppression of eddy current resulting from the lower electric conductivity of GO-FeSiAl compared to pure FeSiAl [41].

**Figure 7:**
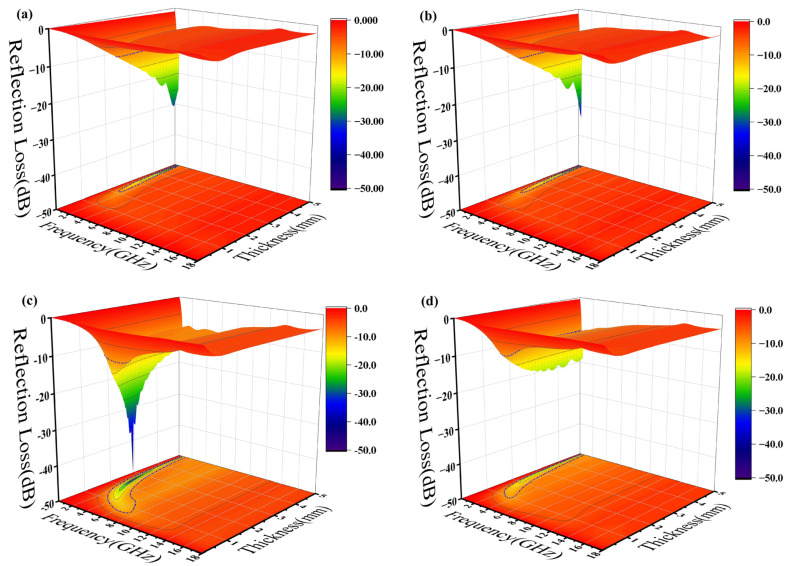
Three-dimensional (3D) projections of reflection loss of epoxy composites with (**a**) GF-1, (**b**) GF-2, (**c**) GF-3, and (**d**) GF-4.

**Figure 8:**
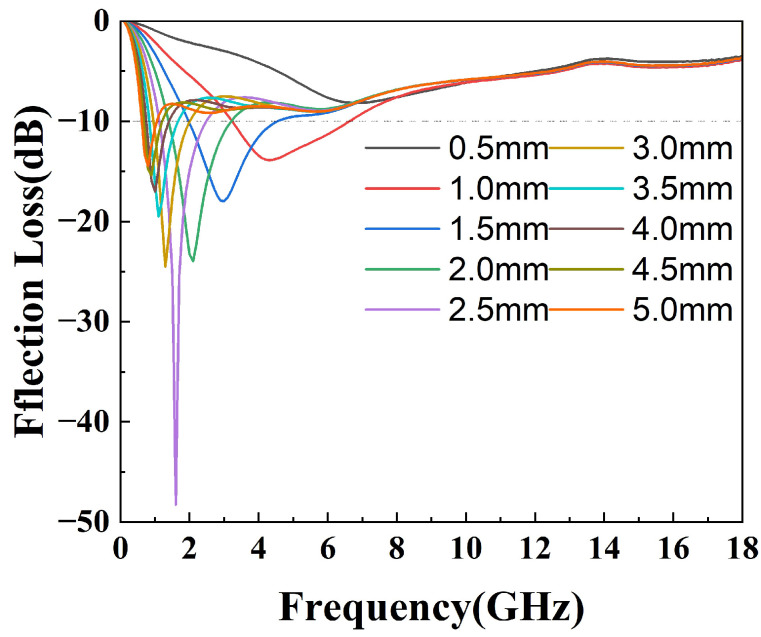
Reflection loss of the epoxy compound with GF-3 at different thicknesses.

According to the transmission line theory, $\varepsilon_{r}$ ($\varepsilon_{r}=\varepsilon^{\prime }-j\varepsilon^{\prime \prime }$) represents relative complex permittivity and $\mu_{r}$ ($\mu_{r}=\mu^{\prime }-j\mu^{\prime \prime }$) represents relative complex permeability. The reflection loss properties (RL) of the GO/FeSiAl epoxy compound can be calculated from $\varepsilon_{r}$ and $\mu_{r}$ through the following equations:

(1) $$RL=20\mathrm{lg}\left|\frac{z_{in}-z_{0}}{z_{in}+z_{0}}\right|$$

(2) $$Z_{in}=Z_{0}\sqrt{\frac{\mu_{r}}{\varepsilon_{r}}}\tan\left(j\frac{2\pi fd}{c}\sqrt{\mu_{r}\varepsilon_{r}}\right),$$

where $Z_{in}$ is the normalized impedance of the sample, $Z_{0}$ is the free space impedance, f is the frequency of the incident microwave, c is the velocity of light in free space, and d is the thickness of the testing sample. The 3D projections of the reflection loss of the epoxy composites from 0 mm to 5 mm thickness and of the 0 GHz to 18 GHz frequency variation are shown in Figure 7. The black contour range of the effective bandwidth indicates an RL value below −10 dB (90% absorption). All the epoxy composites have a fairly wide area to within this standard. In the sample with pure silylated FeSiAl (Figure 7a), the maximum reflection loss is −30.75 dB at 0.5 GHz and at a 4.95 mm thickness, with an efficient bandwidth of 0.4 GHz. While, as shown in Figure 7b, with only 0.1% of GO amount in the FeSiAl system, the maximum reflection loss increases by 10% to −34.00 dB at 0.5 GHz, and the effective bandwidth increases by 20% to 0.5 GHz. However, epoxy compounds with pure magnetic particles or a low GO amount show fairly narrow effective bandwidths, and strong absorption peaks only fall in a very low frequency band. As shown in Figure 7c for the GF-3 sample with 1% of GO, the maximum reflection loss increases to −48.29 dB at 1.6 GHz and the effective bandwidth increases to 3.8 GHz, which exhibits a wider bandwidth (3.8 GHz vs. 3.12 GHz [42] and 3.52 GHz [43]) and a higher refection loss (−48.29 dB vs. −47 dB [44], −44.47 dB [42] and −48.08 dB [39]) in the most recent relevant literature, using FeSiAl as a microwave absorber. This provides a perfect match for 5G station application, as the effective bandwidth just falls in the range of the 5G antennas’ operating frequency band in the 2.575–2.645 GHz range [45], as shown in Figure 8. It can be concluded that, in most cases, the microwave reflection loss increased greatly as the GO amount increased. However, it decreases as the amount of GO reaches 10% in GF-4, as shown in Figure 7d. This might result from the decrease of complex permittivity. Overall, controlling the amount of GO in GO/SiFeAl epoxy compounds can facilely adjust the dissociation and frequency of the absorption peak.

Factors affecting the performance of wave absorption also include impedance matching and the attenuation constant. Generally, the closer the value of impedance matching Z (Z = $Z_{in}/Z_{0}$) is to 1, the more the incident electromagnetic wave can enter the material without being emitted. Another parameter reflecting the attenuation characteristics is the attenuation constant (α), which can be calculated through the following equation:

(3) $$\alpha =\frac{\sqrt{2}\pi f}{c}\sqrt{\left(\mu^{\prime \prime }\varepsilon^{\prime \prime }-\mu^{\prime }\varepsilon^{\prime }\right)+\sqrt{{\left(\mu^{\prime \prime }\varepsilon^{\prime \prime }-\mu^{\prime }\varepsilon^{\prime }\right)}^{2}+{\left(\mu^{\prime }\varepsilon^{\prime \prime }+\mu^{\prime \prime }\varepsilon^{\prime }\right)}^{2}}}.$$

**Figure 9:**
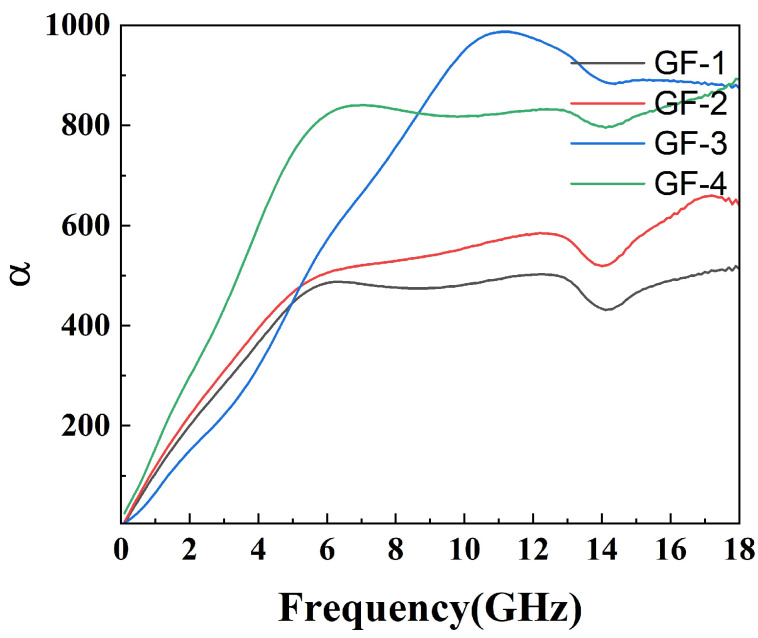
Attenuation constant (α) of epoxy compounds.

**Figure 10:**
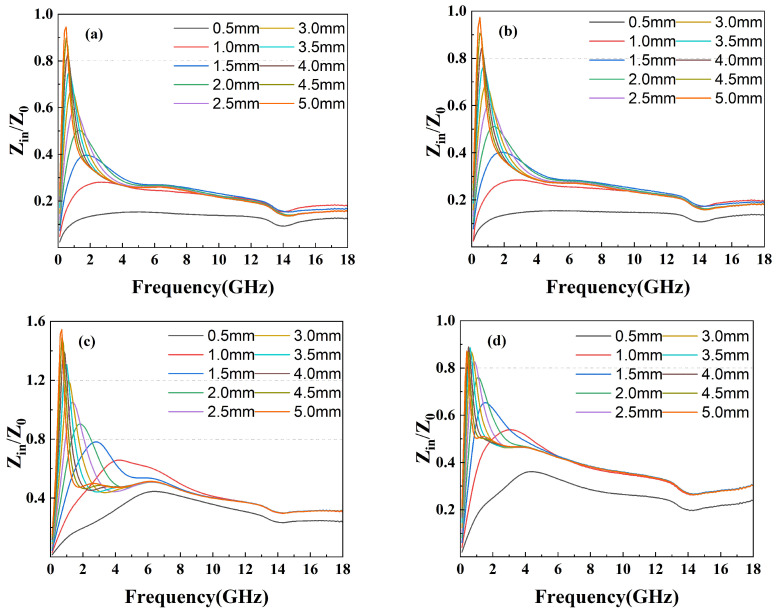
Impedance matching Z of epoxy composites with (**a**) GF-1, (**b**) GF-2, (**c**) GF-3, and (**d**) GF-4.

The attenuation constant reflects the material’s ability to dissipate electromagnetic waves. However, a strong attenuation constant often leads to low impedance matching, so it is necessary to balance the effects of both. The attenuation constant of the compounds is shown in Figure 9 while the impedance matching is shown in Figure 10. As shown in Figure 9, the attenuation constant increases as the amount of GO grows, indicating more electromagnetic energy loss resulting from the porous structure and defects on the surface of GO. However, GF-4 with the largest GO amount and the highest attenuation constant shows a low reflection loss due to its low impedance matching as shown in Figure 10d, which is consistent with the conclusion from the reflection loss results. In conclusion, 1% of GO is a reasonable amount among others that reaches a balance between magnetic loss and conductive loss.

**Figure 11:**
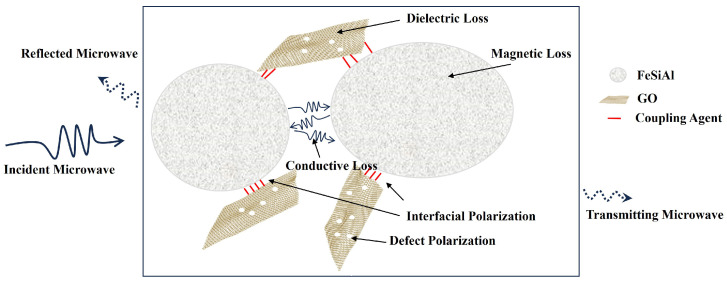
Schematic of the GO/FeSiAl epoxy compounds microwave absorption mechanism.

Figure 11 is the schematic of the GO/FeSiAl microwave absorption mechanism. To begin with, GO and FeSiAl can perform as wave-absorbing agents separately. GO has a high dielectric loss from defect polarization and dipole polarization, while FeSiAl has a high magnetic loss from eddy current loss at a low frequency and nature resonance at a higher frequency. Then, since GO and FeSiAl have different polarity and conductivity, interfacial polarization occurs at the interface between GO and FeSiAl, which contributes greatly to the microwave dielectric loss. There is also conductive loss between FeSiAl interfaces. Finally, GO and FeSiAl have a synergistic effect as, at a high frequency, GO can lower FeSiAl’s permeability and prevent a skin effect, therefore forming a better impedance matching. It can be concluded that the GO/FeSiAl epoxy compounds have strong absorption and a broad effective bandwidth covering the operating frequency of 5G antennas with a thin coating so that it could provide a potential means for efficient microwave absorption for a 5G base station.

### 3.3. Thermal Conductivity Analysis

**Figure 12:**
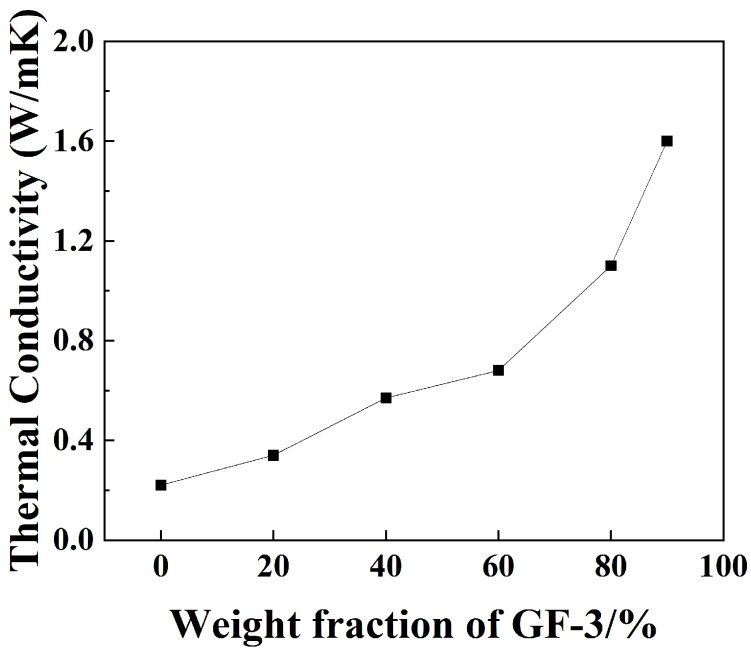
Thermal conductivity analysis of epoxy compounds with different ratios of GF-3.

Figure 12 shows the thermal conductivity of GF-3/epoxy compounds in different weight fractions. The thermal conductivity grows as the weight fraction increases, and a sudden acceleration of growth occurs at 60%. This is because the epoxy matrix’s thermal conductivity is very low compared to the filler itself. When the fraction of filler is low, the epoxy matrix acts as a thermal barrier positioned between fillers [46]. When the filler fraction reaches a certain point, a thermal channel is formed. The maximum result of 1.6 W/(mK) was obtained when the weight fraction of GF-3 in epoxy compounds was 90%, which was 605% higher than that of pure epoxy, respectively.

## 4. Conclusions

Our work provides a simple and practical way to fabricate multifunctional 5G base station packaging materials comprising silylated GO/FeSiAl/epoxy, which can be realized by using a simple solvent-handling method. It could act as a small thickness microwave absorber within a range of operating frequency for the 5G antennas of 2.575–2.645 GHz, as evidenced by a reflection loss with a maximum of −48.28 dB and an effective range of 3.6 GHz. Such a prominent microwave absorbing performance results not only from the interfacial polarization reasonance attributed to the nicely formed GO/FeSiAl interface but also the synergistic effect from excellent impedance matching by GO compromising FeSiAl’s high conductivity at a high frequency. It also exhibits a significantly enhanced thermal conductivity of 1.6 W/(mK), performing a remarkable heat conducting ability by constructing successive thermal channels. All in all, the GO/FeSiAl epoxy compounds show promising results of strong absorption and a broad effective bandwidth covering the operating frequency of 5G antennas with a thin coating so that it could provide potential efficient microwave absorption for a 5G base station.

## Figures and Tables

**Table 1:** The composition of the samples.

Samples	Coupling Agent Content (mg/mL)	Mass Ratio of FeSiAl (%)	Mass Ratio of GO (%)
GO-1	0.2	0	1
GF-0	0	99	1
GF-1	0.2	100	0
GF-2	0.2	99.9	0.1
GF-3	0.2	99	1
GF-4	0.2	90	10

## Data Availability

Data are contained within the article.
